# Low cost multifunctional 3D printed image quality and dose verification phantom for an image-guided radiotherapy system

**DOI:** 10.1371/journal.pone.0266604

**Published:** 2022-04-06

**Authors:** Jian-Kuen Wu, Min-Chin Yu, Shih-Han Chen, Shu-Hsien Liao, Yu-Jen Wang

**Affiliations:** 1 Division of Radiation Oncology, Departments of Oncology, National Taiwan University Hospital, Taipei, Taiwan; 2 Department of Radiation Oncology, Taipei Medical University Hospital, Taipei, Taiwan; 3 Department of Medical Imaging, National Taiwan University Cancer Center, Taipei, Taiwan; 4 Institute of Electro-Optical Science and Technology, National Taiwan Normal University, Taipei, Taiwan; 5 Department of Radiation Oncology, Fu Jen Catholic University Hospital, New Taipei City, Taiwan; 6 School of Medicine, College of Medicine, Fu Jen Catholic University, New Taipei City, Taiwan; University of Nebraska Medical Center, UNITED STATES

## Abstract

**Purpose:**

Image-guided radiation therapy (IGRT) is used to precisely deliver radiation to a tumour to reduce the possible damage to the surrounding normal tissues. Clinics use various quality assurance (QA) equipment to ensure that the performance of the IGRT system meets the international standards set for the system. The objective of this study was to develop a low-cost and multipurpose module for evaluating image quality and dose.

**Methods:**

A multipurpose phantom was designed to meet the clinical requirements of high accuracy, easy setup, and calibration. The outer shell of the phantom was fabricated using acrylic. Three dimensional (3D) printing technology was used to fabricate inner slabs with the characteristics of high spatial resolution, low-contrast detectability, a 3D grid, and liquid-filled uniformity. All materials were compatible with magnetic resonance (MR). Computed tomography (CT) simulator and linear accelerator (LINAC) modules were developed and validated.

**Results:**

The uniformity slab filled with water is ideal for the assessment of Hounsfield units, whereas that filled with wax is suitable for consistency checks. The high-spatial-resolution slab enables measurements with a resolution up to 5 lp/cm. The low-contrast detectability slab contains rods of 5 different sizes that can be clearly visualised. These components meet the American College of Radiology (ACR) standards for QA of CT simulators and LINACs.

**Conclusions:**

The multifunctional phantom module meets the ACR recommended QA guidelines and is suitable for both LINACs and CT-sim. Further measurements in an MR simulator and an MR linear accelerator (MR-LINAC) will be arranged in the future.

## I. Introduction

With the rapid development of radiation therapy, the use of cone-beam computed tomography (CBCT) and/or magnetic resonance imaging (MRI) for image guidance has become a routine practice in modern radiation therapy [1, 2]. Several sophisticated image-guided techniques are being used for advanced or adaptive external beam radiotherapy [3]. These require highly accurate geometric and volumetric anatomic imaging information to reduce the internal and setup margins around the planning target volumes to achieve high performance in local tumour control while maintaining a low risk of normal tissue complications [4]. To optimally utilise the information provided by commercial image-guided radiation therapy (IGRT) systems, such as CT-sim, MR-sim, kilovoltage cone-beam computed tomography (kV CBCT), and megavoltage cone-beam computed tomography (MV CBCT) performing quality assurance (QA) of the imaging system is required [1, 4, 5]. Phantoms such as the Gammex American College of Radiology (ACR) computed tomography (CT) accreditation phantom and ACR MRI phantom are commercially available; however, they can be expensive for some hospitals, particularly for those in developing countries. The objective of this study was to develop a low-cost and multipurpose module for evaluating image quality and measuring the dose using an ionisation chamber for dose verification. We consider the material used in this study to be of “low cost” in the sense that the material can be easily obtained locally for a few hundred U.S. dollars. The proposed low-cost in-house phantom is compatible with CT, MRI, and a linear accelerator (LINAC) for various performance tests.

## II. Materials and methods

### A.) Phantom module design

**Fig 1:**
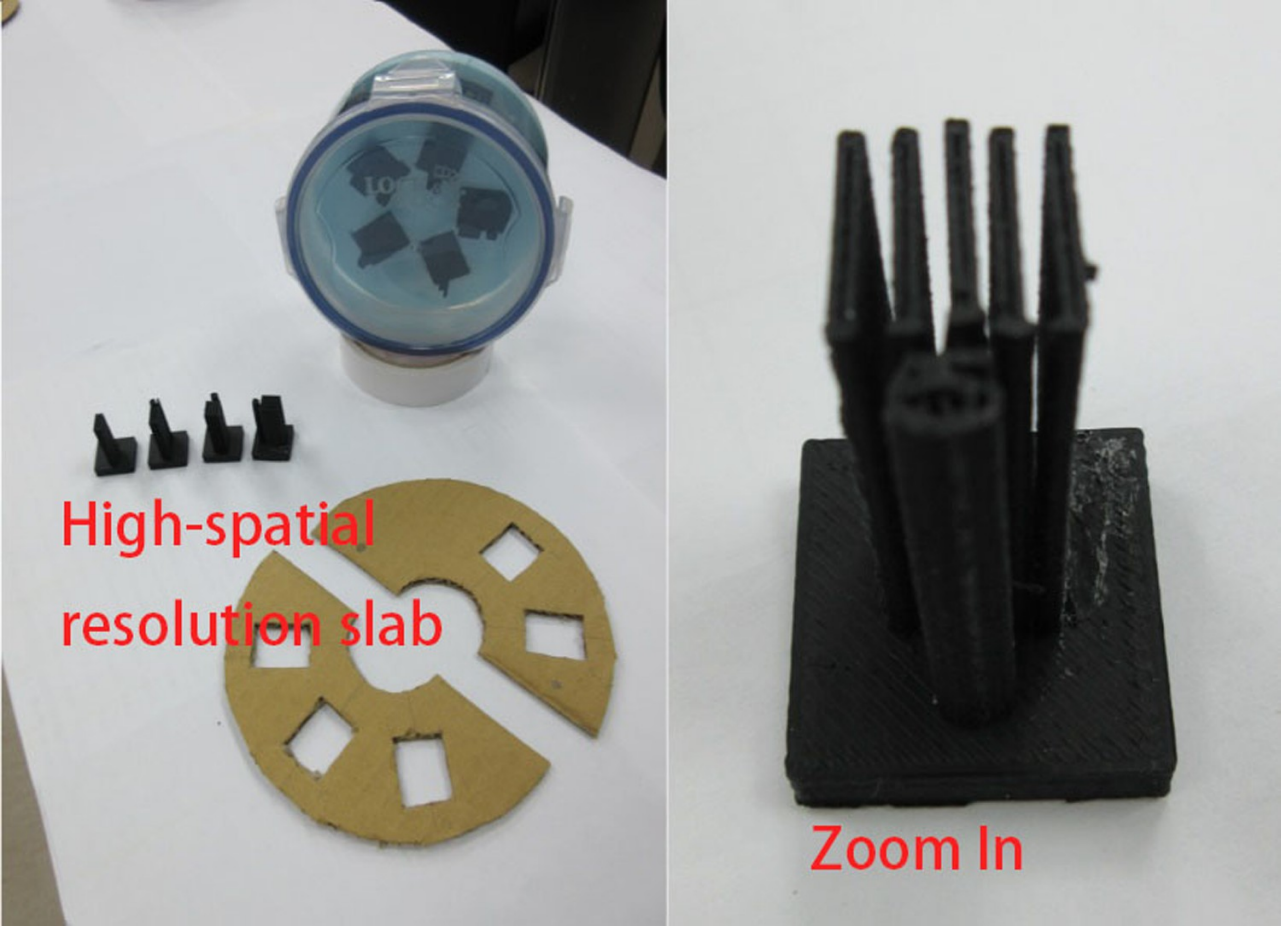
High-spatial-resolution slab printed using a three-dimensional (3D) printer.

**Fig 2:**
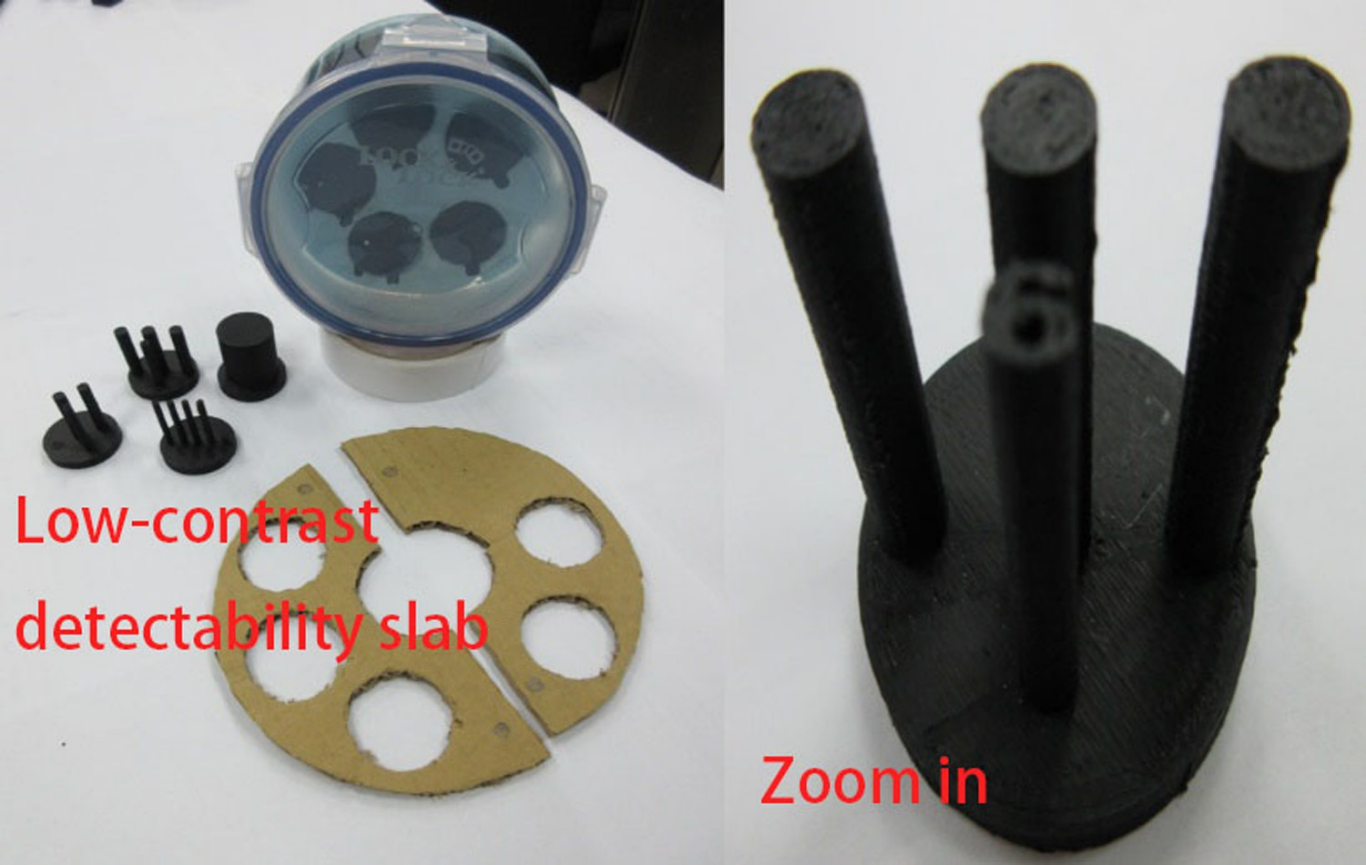
Low-contrast detectability slab.

**Fig 3:**
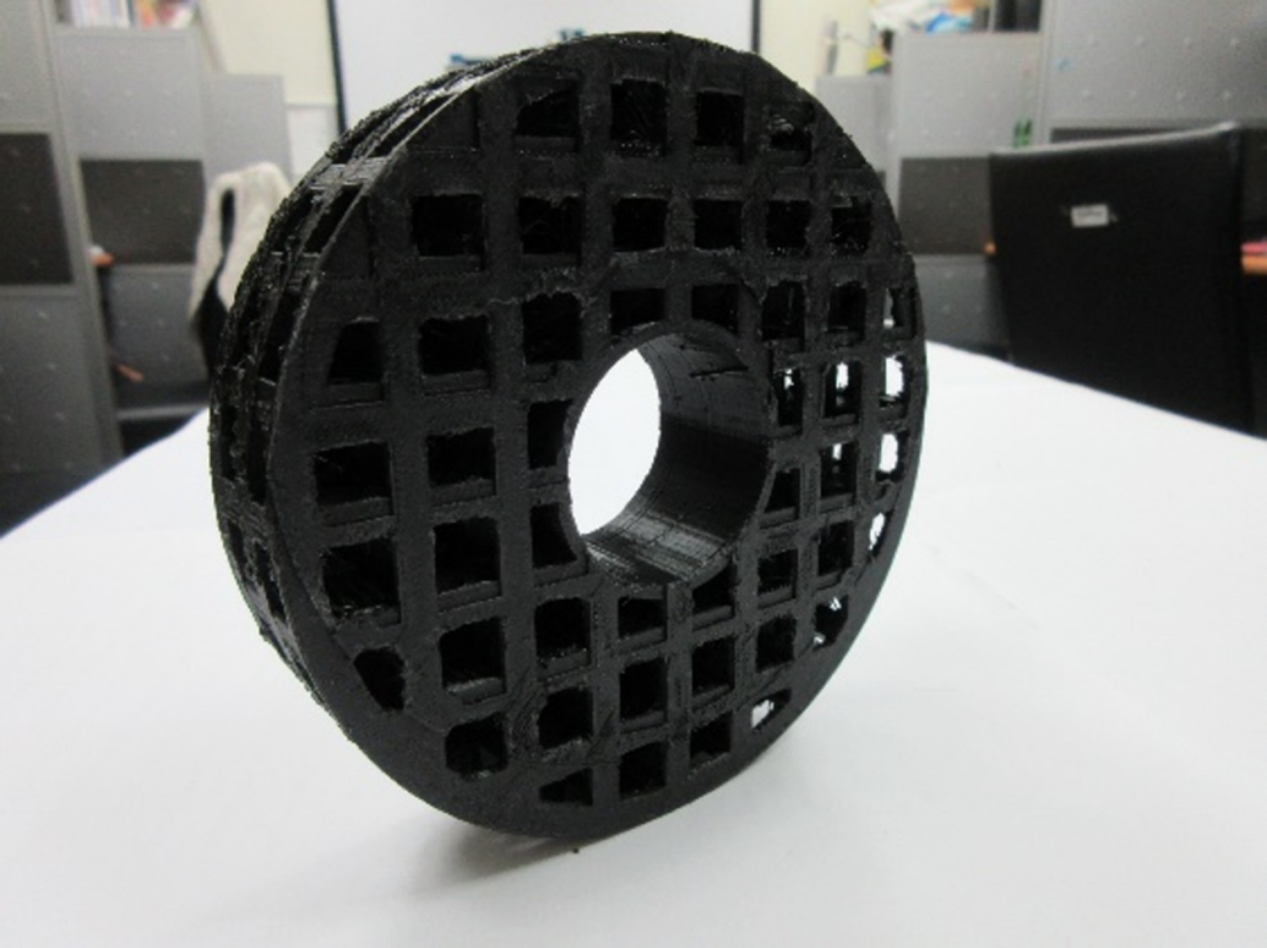
Slab with a 3D grid with equal spacing.

The proposed phantom module includes a high-spatial-resolution slab (Fig 1), a low-contrast detectability slab (Fig 2), a slab with a three-dimensional (3D) grid with equal spacing (Fig 3), and a uniform liquid-filled slab. For the present study, specimens were manufactured using the desktop 3D printer FlashForge Creator Pro BEAVER III (Mastech Machine Co. Ltd., New Taipei City, Taiwan), the inkjet printing 3D printer by moving the nozzle in the x-y direction while extruding melted material. The minimum printing layer thickness of this machine is 0.1 mm, the nozzle can be heated up to 230°C degrees, and the positioning precision in the z-axis and x-y axis are 0.0025 mm and 0.011, respectively. The maximum print area can be up to 225 x 145 x 155mm^3^, polylactic acid (PLA), and dissolvable material are acceptable materials and the suitable working temperature is 228 to 230°C according to the information provided from its official website [6]. Filaments made by PLA were chosen for the suitable operable temperature range of our 3D printer. The filament control was aluminium 6061 Filamet^TM^. The uniformity slab could be filled with water, wax, or silicone oil, depending on the imaging system being used. Each cylindrical slab was made of acrylic and had a diameter of 20 cm and a thickness of 8 cm to represent the head, and a hole passed through the centre of the phantom to insert a farmer-type chamber to check the consistency of the output. The printing materials were mostly composed of Proto-pasta PLA (BCN3D Technologies, Barcelona, Spain) and beeswax. For CT simulation (CT-sim) or CBCT, PLA may be applicable. However, an Al-Si-Mg alloy is necessary if the design is applied to MR-sim or MR-LINAC. The module we show here that was mainly used in CT-sim or CBCT consisted primary of PLA.

### B) Computed tomography simulator module

**Fig 4:**
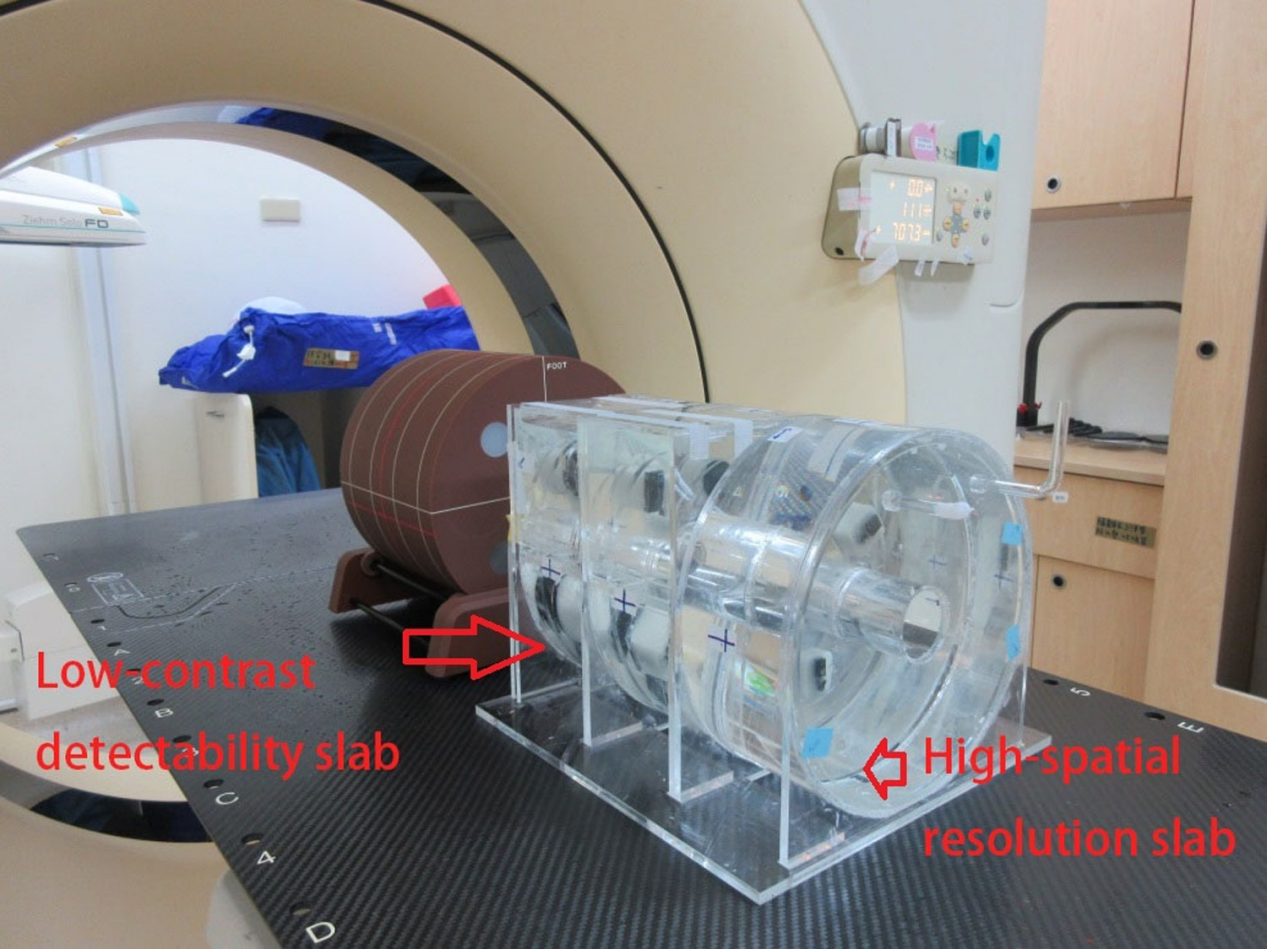
Computed tomography (CT) simulator module: A. high-spatial-resolution slab; B. low-contrast detectability slab; C. water-filled uniformity slab.

The CT image quality depends on the contrast resolution, spatial resolution, image noise, and artefacts [7]. To facilitate the QA of CT images, a high-spatial-resolution slab, a low-contrast detectability slab, and a liquid-filled uniformity slab were selected as a self-contained unit. The high-contrast-resolution slab offers resolution patterns of 4, 5, 6, 7, and 8 line pairs per centimetre (lp/cm), and the low-contrast detectability slab provides several cylindrical rods with 3, 4, 5, 6, and 25 mm diameters. The widths of the bars and spaces were equal. Plastics of various densities between 0.3 and 1.2 g/ml were used to fabricate the CT phantom line-pair patterns using a 3D printer. The phantom module is depicted in Fig 4.

The uniformity slab of the phantom was filled with three different materials, and the filled phantom was scanned with the same parameters during each scan to ensure uniform exposure conditions. Images were taken with a Philips Brilliance Big Bore (BBB) CT scanner using an X-ray tube voltage of 120 kV and a current of 650 mA. Helical CT scans were performed using a 1.5 mm slice thickness. Each material was scanned 3 times under the same conditions.

### C) Magnetic resonance imaging module

**Fig 5:**
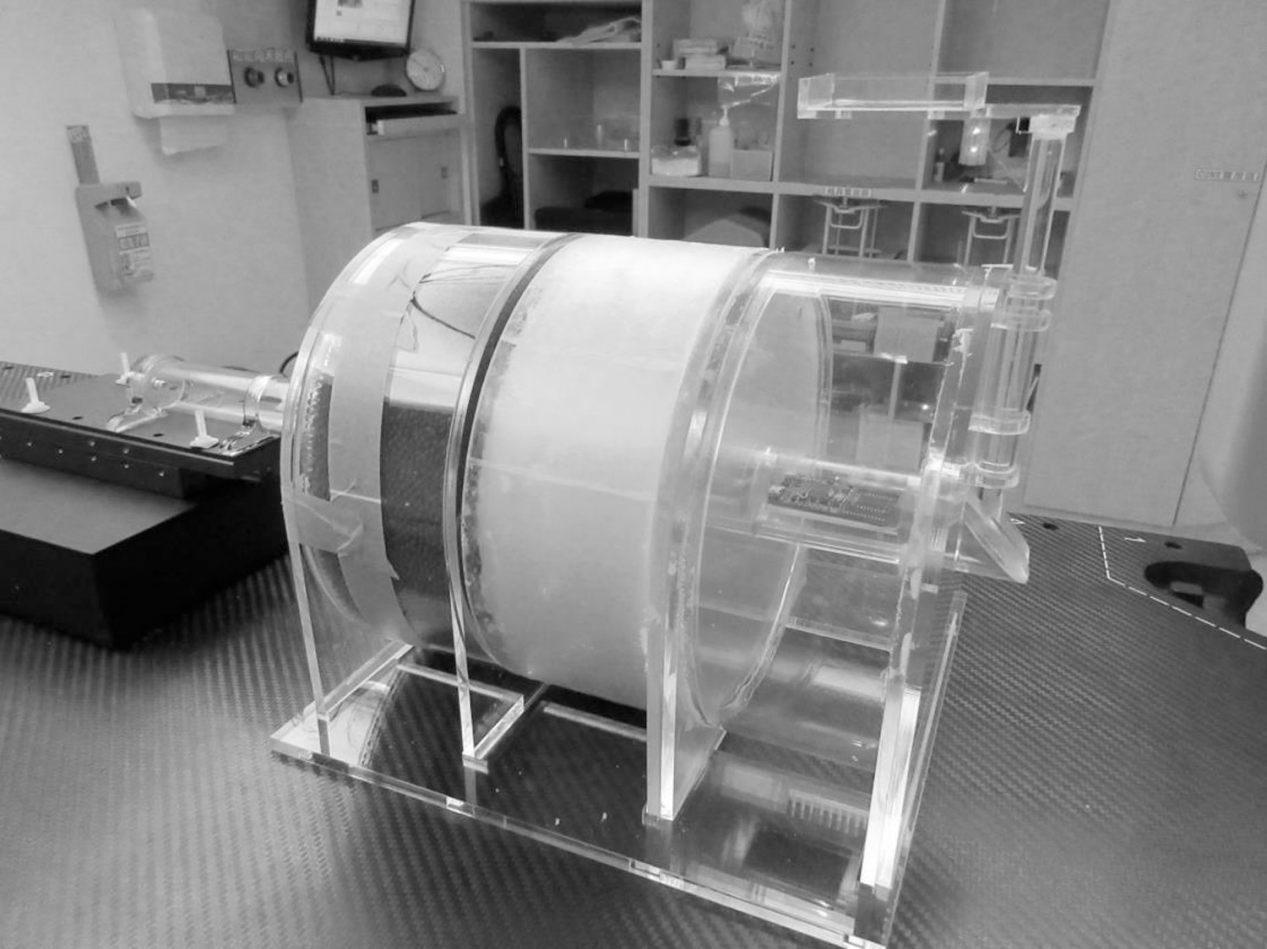
Magnetic resonance imaging module connected to a piezoelectric motor.

The MRI module of the phantom can be used in both MRI and LINACs. It is composed of slab with a 3D grid with equal spacing and an MR signal-generating liquid-filled slab for distortion checks (Fig 5). The slab centre was drilled to accommodate an ion chamber insert, which was connected to a nonferromagnetic piezoelectric motor as a motion management QA phantom or to keep the chamber in the centre for an output consistency check.

### D) Linear accelerator module

**Fig 6:**
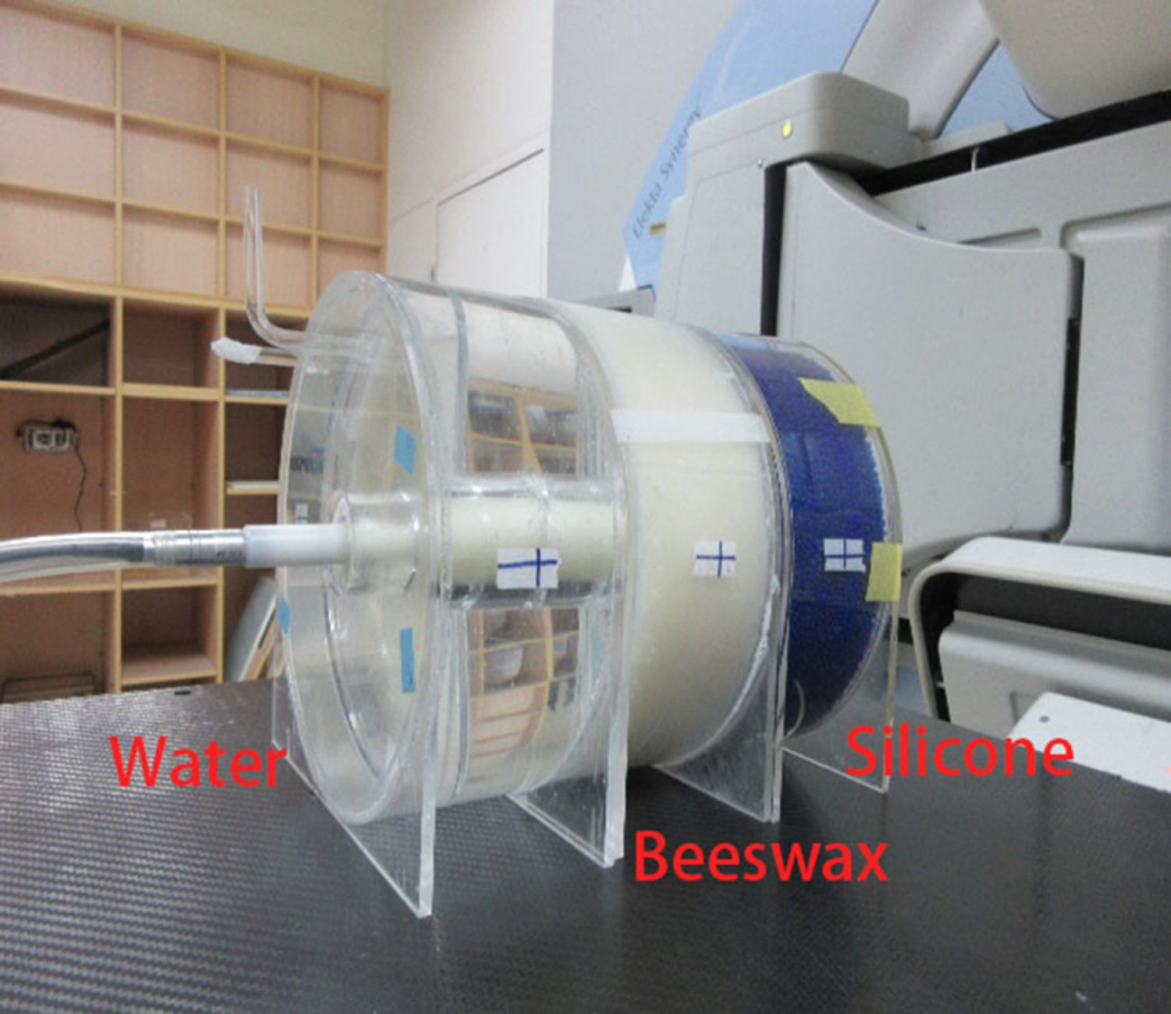
Inserting the ion chamber in the centre cavity for dose verification.

**Fig 7:**
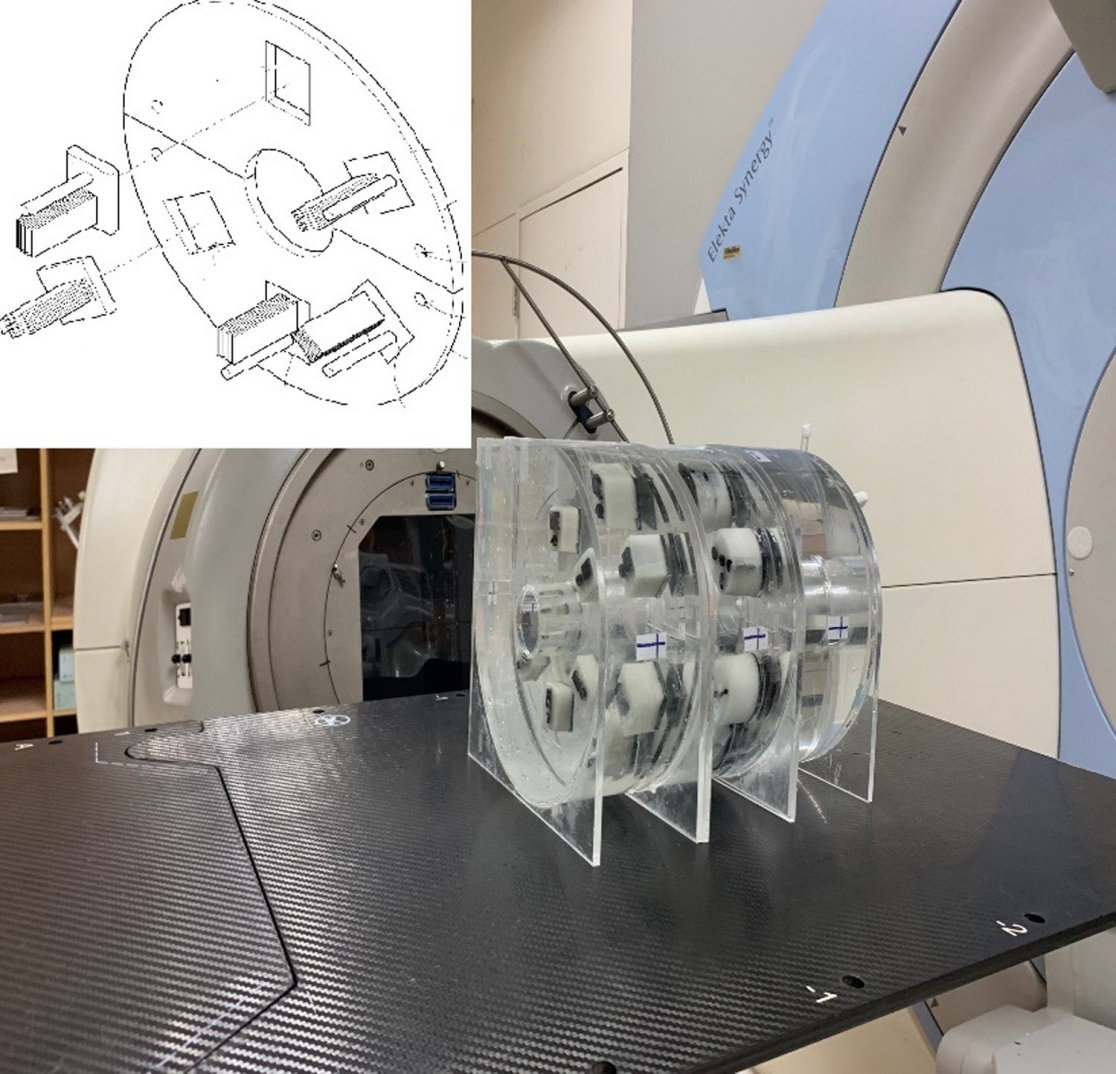
Image quality module.

The LINAC module provides a dual function and can be used for both dose verification and image quality assessment of CBCT systems integrated with a radiotherapy machine. Similar to the design in the MR module, a Farmer-type chamber is inserted in the centre of the liquid-filled slab for dose verification (Fig 6). For image quality assessment, the use of the same settings as in the CT simulator module ensures balance between optimising the image quality and minimising the radiation dose (Fig 7). A Mitutoyo vernier calliper with 0.01 mm accuracy was used for QA to confirm the dimensions of the 3D printed items.

## III. Results

The total cost of the multifunctional phantom used in this experiment was less than 1,000 US dollars (failed parts not counted), and approximately 4 hours were needed to complete the printing process. All images were exported in DICOM format for offline analysis.

### III.I Computed tomography simulator module

**Fig 8:**
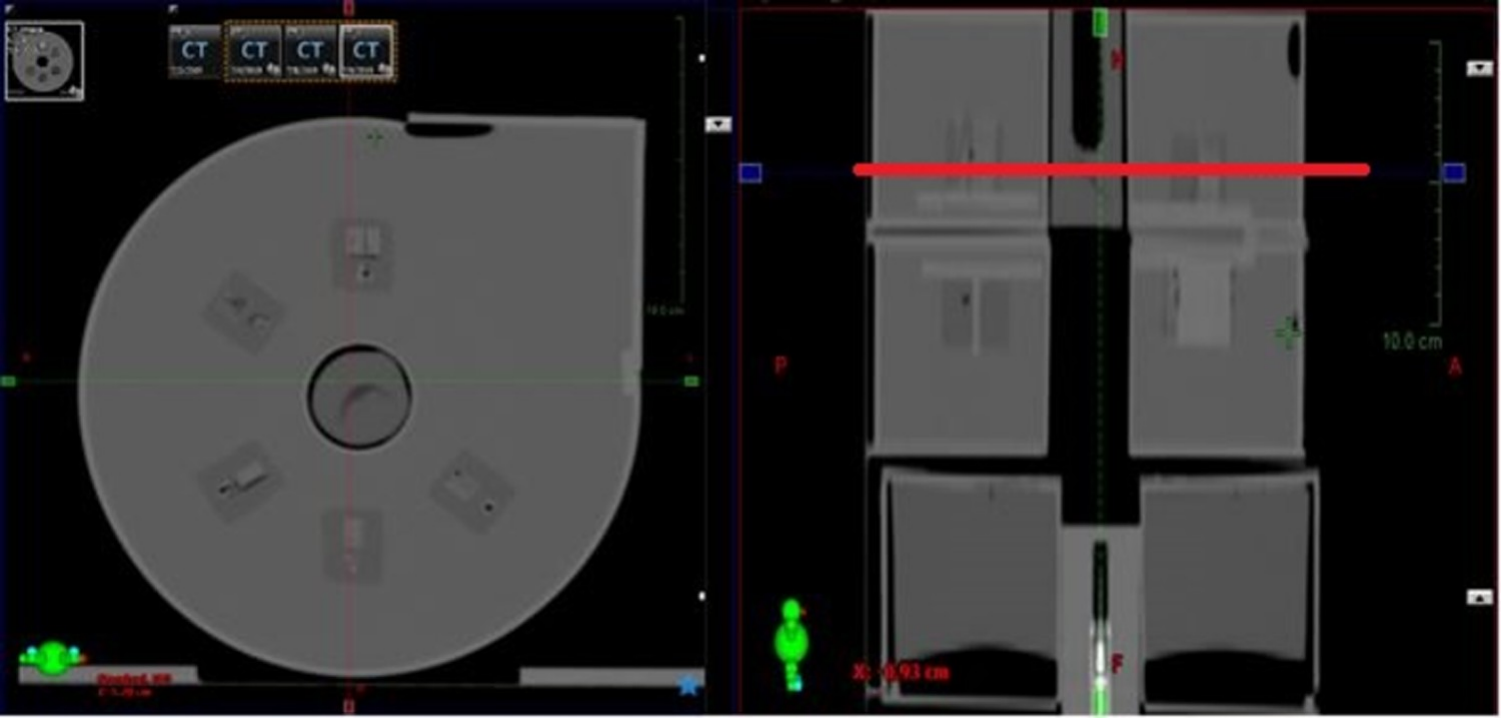
CT image of the high-spatial-resolution slab.

The tolerance limits for the spatial resolution and contrast resolution were set according to manufacturer specifications (Philips CT BBB) and ACR recommendations. The goal of this phantom module was to meet the ACR passing criteria because the CT ACR464 Phantom was scanned in similar scenarios. Using adult head and high-resolution chest (HRC) scanning protocols, spatial resolutions of 5 and 6 lp/cm, respectively, should be resolved according to ACR recommendations [8]. The window width and level were optimised for ideal visualisation. Analysing the results of the high-contrast-resolution slab (Fig 8), the limiting spatial resolution of the high-contrast-resolution slab was 5 lp/cm (4 lp/cm was clearly distinguishable).

**Fig 9:**
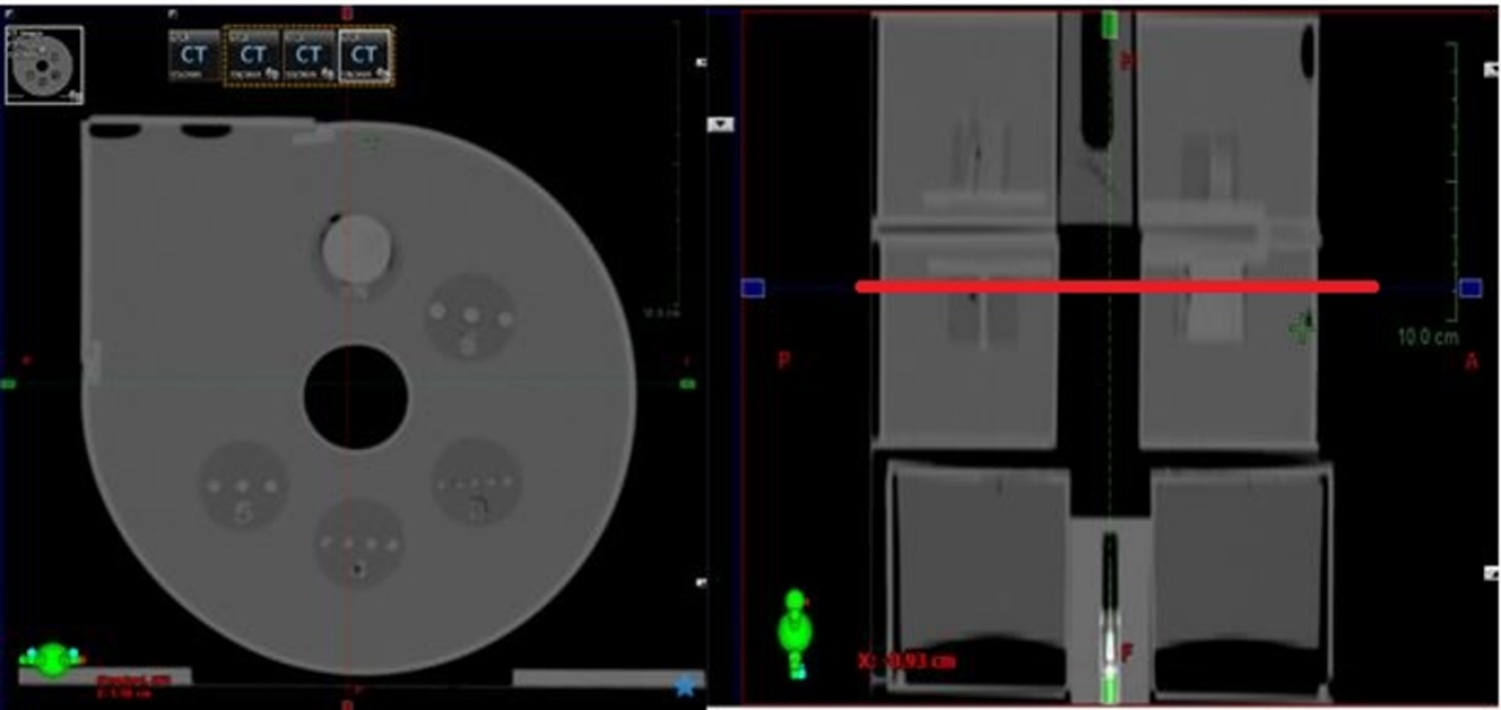
CT image of the low-contrast detectability slab.

For the low-contrast detectability slab, ACR recommends that 6 mm rods should be clearly visible [8]. The slab depicted in Fig 9 contains five sets of cylindrical rods with different diameters (3, 4, 5, 6, and 25 mm). From our analysis, all five sizes of the rods can be visualised from the DICOM image. It can be concluded that the low-contrast detectability slab meets the tolerance recommended by ACR.

For uniformity determination, the uniformity slab was filled with three different materials (namely, water, wax, and silicone oil) and analysed separately. The uniformity data were acquired from the four edges (3, 6, 9, and 12 o’clock); the ACR recommends the uniformity tolerance to be less than 5 HU for all four edge positions, and the centre CT value should be within ±7 HU. Because this phantom had been drilled at the centre for the chamber insert, the value at the centre could not be analysed.

**Fig 10:**
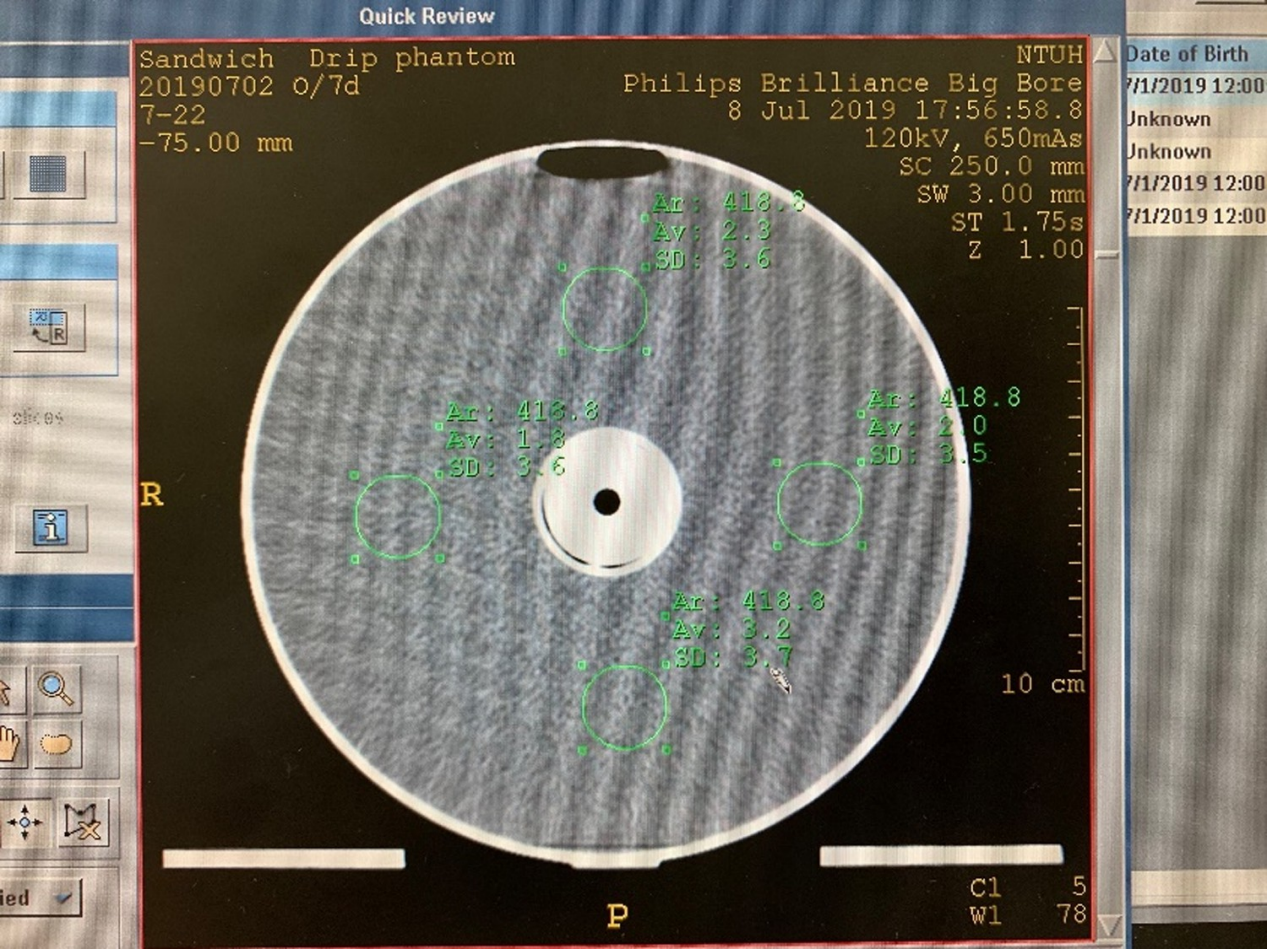
CT image of the water-filled uniformity slab.

Regions of interest (ROIs) of 418.8 mm^2^ were placed at the four edge positions, as shown in Fig 10, when analysing the water-filled uniformity slab. Data analysis results, including the average value across all three tests, are shown in Table 1. The mean CT numbers of tests 1, 2, and 3 at the top, bottom, left, and right were 2.8, 2.2, 2.5, and 2.1, respectively (Table 1). The standard deviation (SD) was approximately 0.31. The largest differences in the mean CT numbers of the four ROIs in tests 1, 2 and 3 were 1.4, 1.3 and 0.5, respectively. which were less than 5 HU. Therefore, this water-filled uniformity slab is assumed to meet the ACR tolerance for uniformity evaluation.

**Table 1:** Summary of the measured CT number using the water-filled uniformity slab.

HU	12 o’clock	3 o’clock	6 o’clock	9 o’clock	Difference	Result
Test 1.	2.3	1.8	3.2	2.0	1.4	PASS
Test 2.	3.2	2.2	1.9	2.1	1.3	PASS
Test 3.	2.8	2.7	2.5	2.3	0.5	PASS
Average	2.8	2.2	2.5	2.1	1.1	

**Fig 11:**
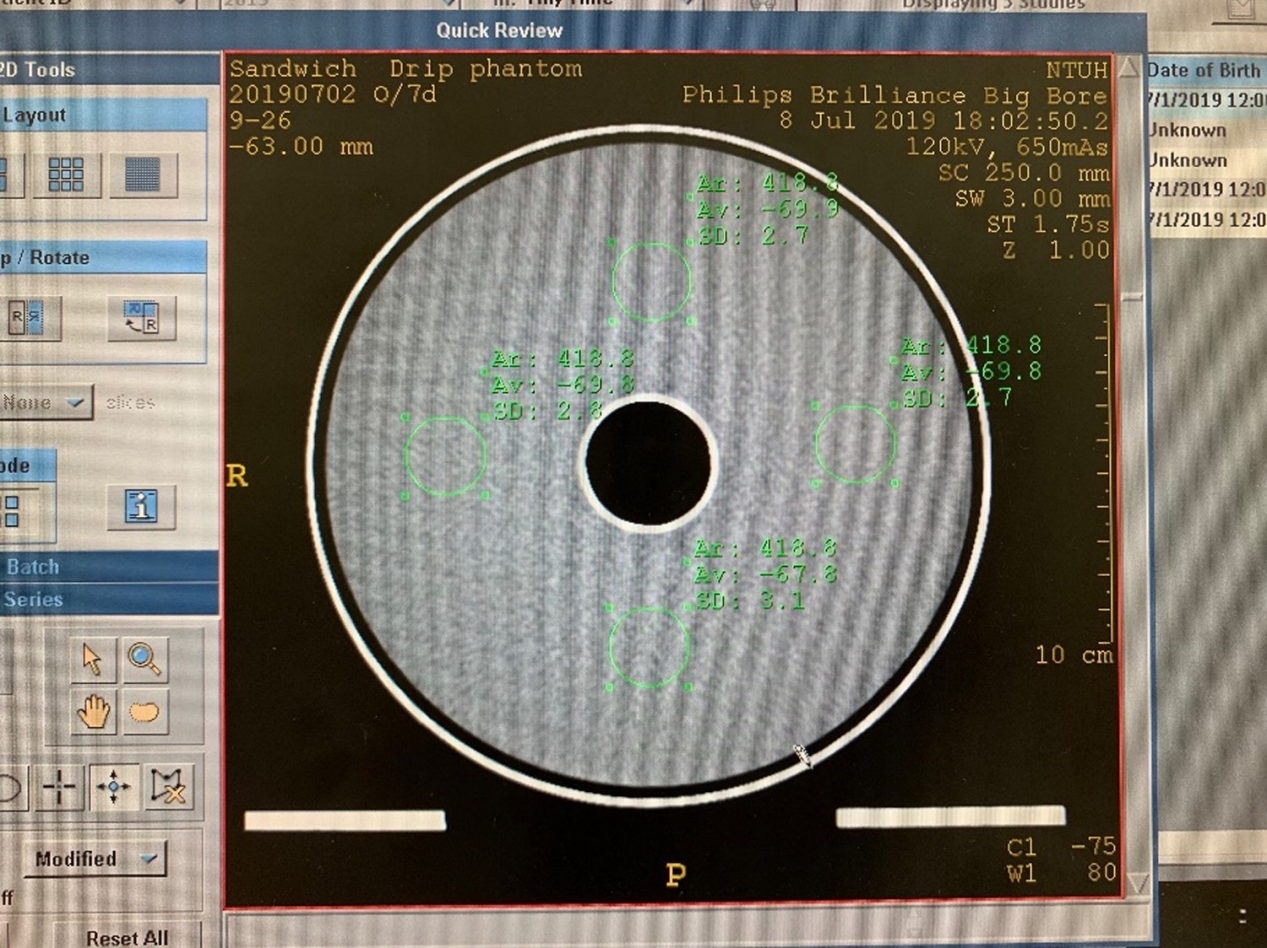
CT image of the wax-filled uniformity slab.

For the wax-filled uniformity slab (Fig 11), the average mean CT numbers across the three tests at the top, bottom, left, and right were -68.7, -66.4, -68.8 and -69.0, respectively (Table 2). The largest differences in the mean CT numbers of the four ROIs were 2.1, 4 and 2.6. This result indicates that the wax can be evenly filled in the cavity for evaluation of the field uniformity. However, it should be noted that the HU of wax is significantly lower than that of true water. Therefore, it is recommended to use the wax-filled slab for a consistency/stability check.

**Table 2:** Summary of measured CT number using the wax-filled uniformity slab.

HU	12 o’clock	3 o’clock	6 o’clock	9 o’clock	Difference	Result
Test 4.	-69.9	-69.8	-67.8	-69.8	2.1	PASS
Test 5.	-69.1	-69.7	-65.7	-69.1	4	PASS
Test 6.	-67.2	-67	-65.6	-68.2	2.6	PASS
Average	-68.7	-68.8	-66.4	-69.0	2.9	

**Fig 12:**
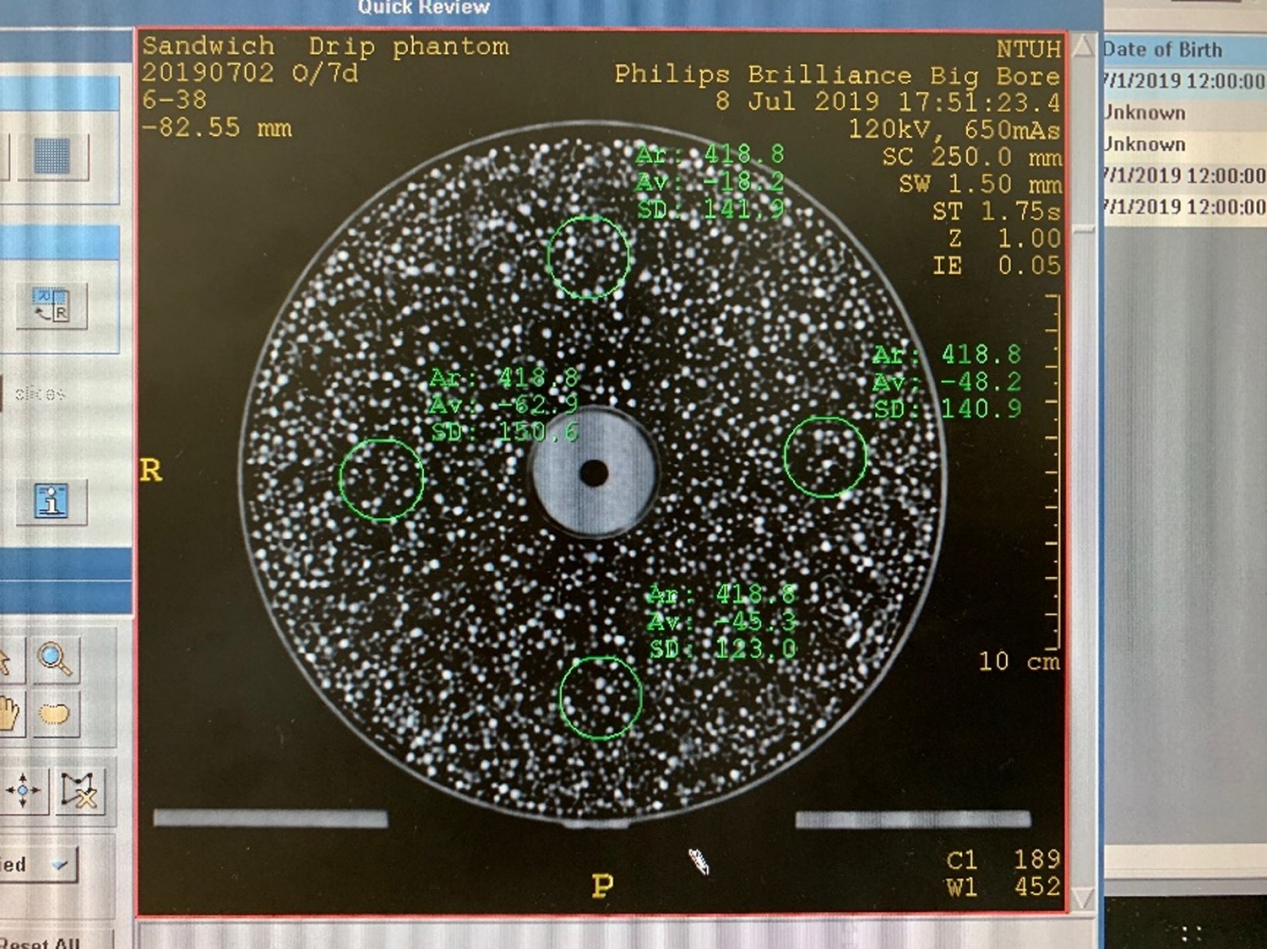
CT image of the silicone-filled uniformity slab.

Finally, the silicone-filled slab was also scanned under the same conditions. Owing to the difficulty in evenly filling the container with silicone, many bubbles and cavities appear in the CT image (Fig 12). The average mean CT numbers across the three tests at the top, bottom, left, and right were -19.8, -43.2, -56.6 and -54.7, respectively (Table 3). These results exceeded the tolerance value, and this material was determined to be unsuitable for filling the uniformity slab.

**Table 3:** Summary of measured CT number using the silicone-filled uniformity slab.

HU	12 o’clock	3 o’clock	6 o’clock	9 o’clock	Difference	Result
Test 7.	-18.2	-62.9	-45.3	-48.2	86	FAIL
Test 8.	-23	-41.4	-44.4	-61.6	38.6	FAIL
Test 9.	-18.2	-59.9	-40.1	-59.9	41.7	FAIL
Average	-19.8	-54.7	-43.2	-56.6	55.4	

### A) Linear accelerator module

**Fig 13:**
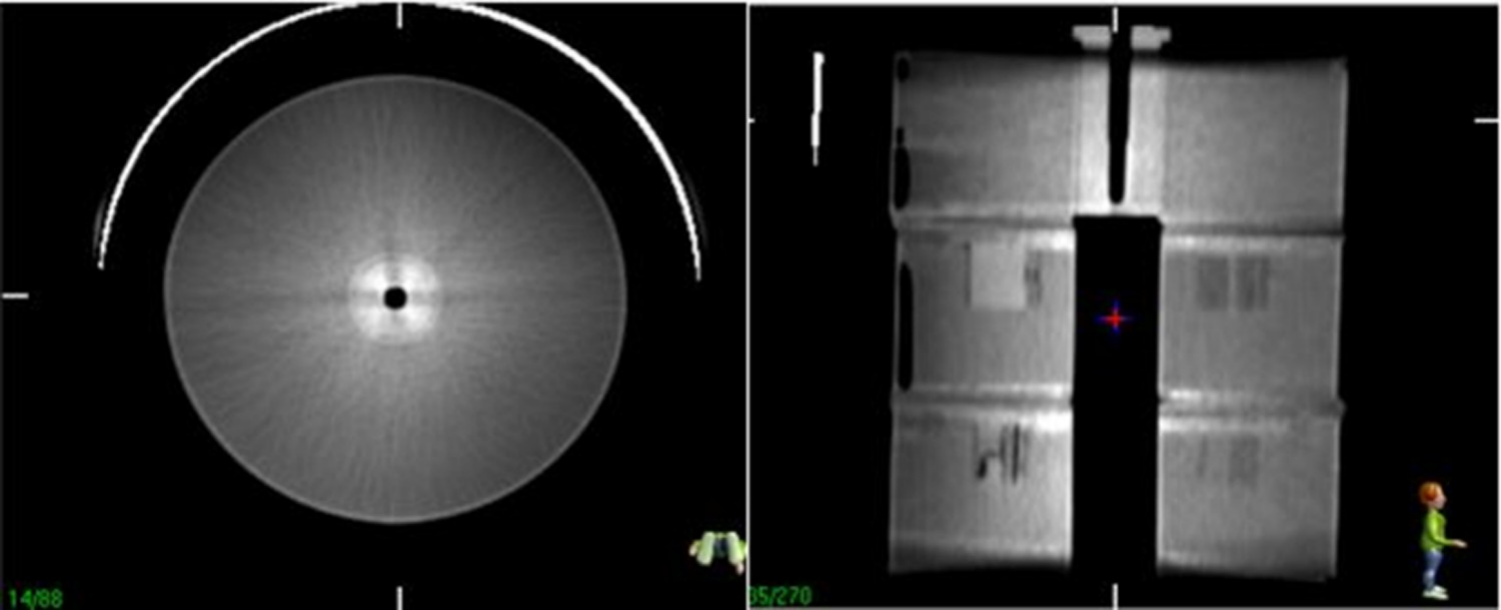
Cone-beam computed tomography (CBCT) images of the water-filled uniformity slab.

Dose verification and image quality evaluation of kV CBCT images were tested using the Elekta Synergy^TM^ XVI system. To verify the dose, a Farmer-type chamber (Capintec Farmer, 0.6 cc PR-06C) was inserted in the centre of the water-filled uniformity slab (Fig 13). The irradiation conditions for 6 and 10 MV were as follows: 200 MU, field size of 6 × 6 cm^2^, and irradiation angles of 0°, 90°, 180°, and 270°. The results of electric charge and ratios are listed in Table 4. The average ratios of the four angles were 0.988 and 0.986 for 6 and 10 MV, respectively, and the SD was 0.5% for both energies (Table 4).

**Table 4:** Analysis of the uniformity slab filled with different materials.

Degree	Water	Wax	Water/wax	Water	Wax	Water/wax
	6 MV (nC)	6 MV (nC)	Ratio	10 MV(nC)	10 MV (nC)	Ratio
**0**	3.093	3.110	0.995	3.370	3.395	0.993
**90**	3.074	3.133	0.981	3.353	3.417	0.981
**270**	3.063	3.105	0.986	3.345	3.399	0.984
**180**	2.995	3.025	0.990	3.283	3.323	0.988
**Average**	3.056	3.093	**0.988**	3.338	3.384	**0.986**
**SD**	0.037	0.041	**0.005**	0.033	0.036	**0.005**

nC: nano coulomb.

For kV CBCT scanning, the same module combination as for the CT-sim QA module was used, namely, the high-contrast-resolution, low-contrast detectability, and water-filled uniformity slabs. The images were acquired using a small collimator cassette, which produced a nominal irradiation field width of 27.67 cm at the isocentre [9].

**Fig 14:**
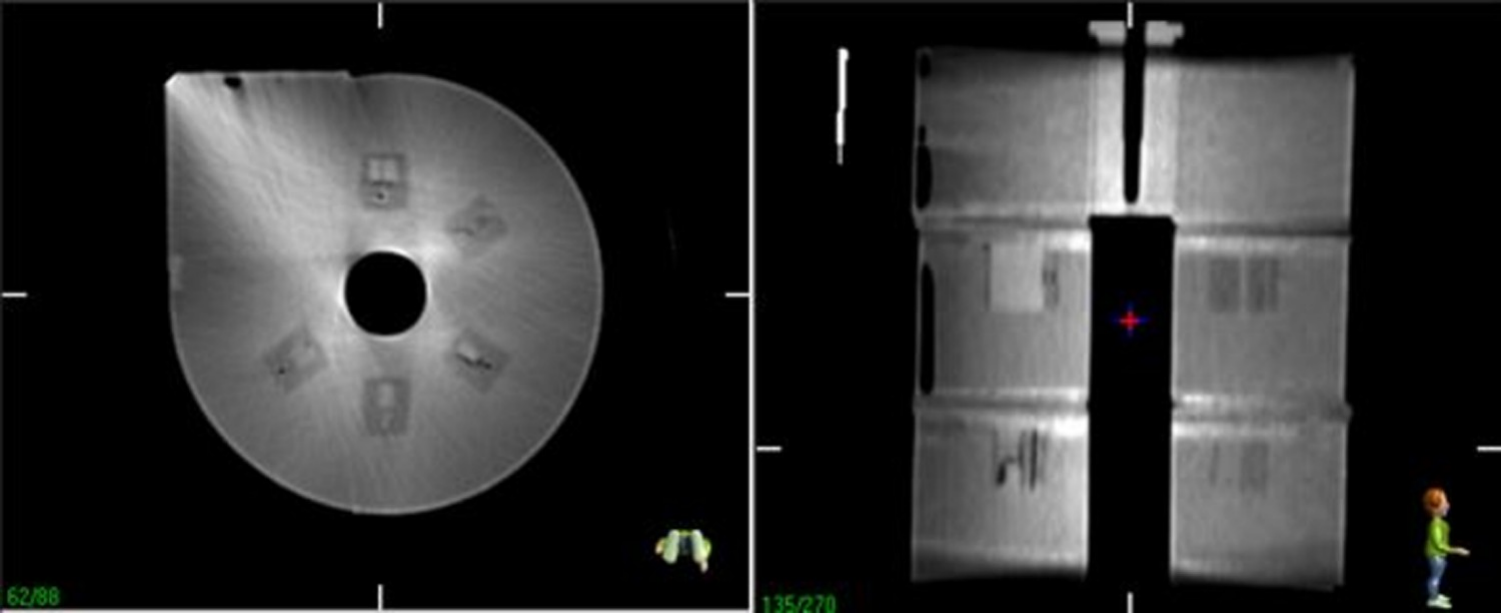
CBCT images of the high-spatial-resolution slab.

Owing to the noise in CBCT images, the high-contrast patterns could not be distinguished from the background, as shown in Fig 14.

**Fig 15:**
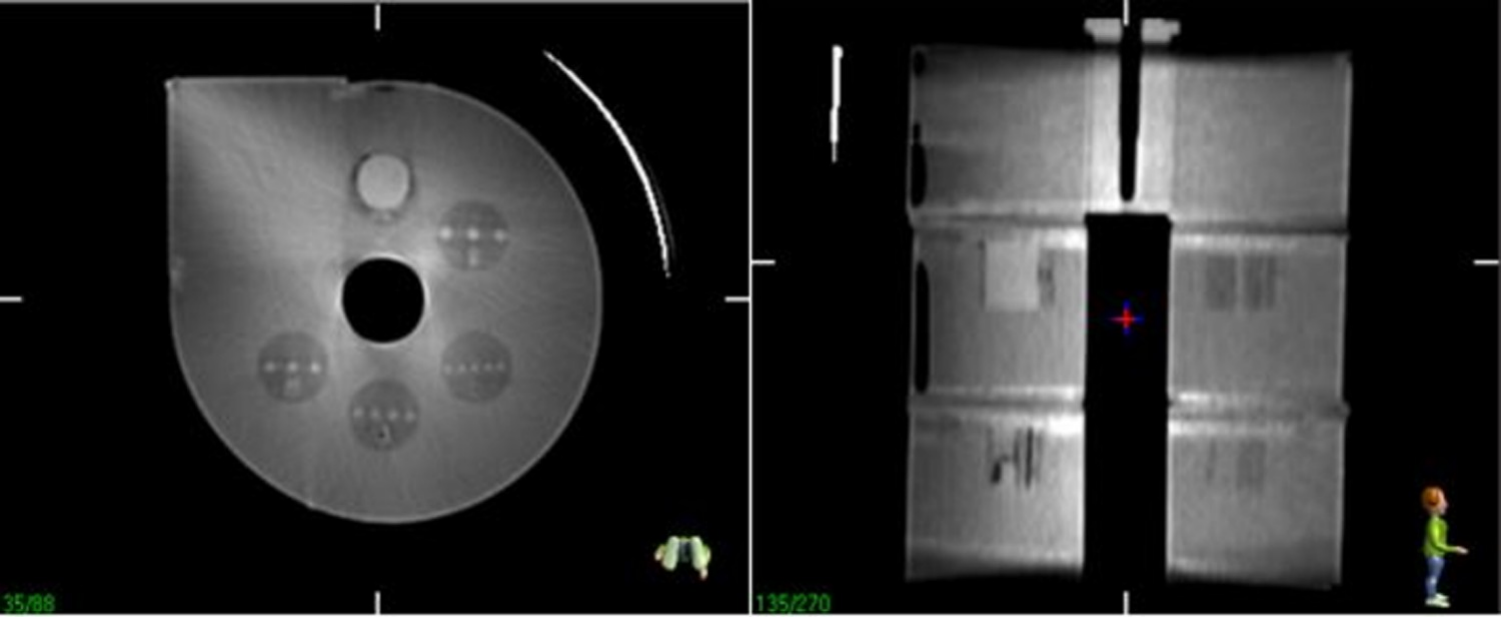
CBCT images of the low-contrast detectability slab.

Note that while the patterns in the high-contrast-resolution slab could not be determined, the low-contrast objects could be clearly resolved (Fig 15).

## IV. Discussion

Compared with other imaging systems, the proposed low-cost and multipurpose module can provide several measurement combinations by choosing different slabs. A Farmer-type chamber is inserted in the centre to verify the dose. In clinical daily practice, this phantom, similar to the ACR phantom, can meet the QA requirement of a CT simulator [8, 10]. For LINACs, similar to the Mobius Verification Phantom™, our innovation can be used to meet the ACR standard [11]. However, our multifunctional innovation has the potential to avoid having to change phantoms frequently, reducing the workload of medical physicists.

Because of the integrated design, easy adjustment is an advantage of our invention. Currently, we need several different types of QA tools to complete the whole QA process. In the future, module QA tools may help accelerate the QA process. Therefore, standard and integrated QA facilities may be an urgent need for clinical usage. Some specific phantoms generated by 3D-printing technologies for dose QA were mentioned before [12], however, to our knowledge, no module QA phantom can perform image QA with similar low cost, and multifunctional advantages to ours.

The cost of this low-cost 3D printed phantom module is only one-tenth that of the commercial CT QA phantoms. Poly (methyl methacrylate)(PMMA) is a common material that is used in RT accessories and phantom parts, thus, the long-term performance of PMMA shells can be considered durable. For the 3D-printed insert, the ageing characteristics show that the hot melt temperature of the 3D printed materials is not lower than that of PMMA. However, in this experiment, the lack of shore hardness and porosity information is a limitation of this experiment. The advantage is that phantoms can be composed according to different needs with higher flexibility. Further study is underway to investigate more physical quantitative methods to achieve the ACR phantom standard.

Beeswax has radiation characteristics similar to those of water, and we can see some clinical will use wax as the bolus to prevent skin sparing effects. Beeswax is very convenient for a moving measurement. Although wax has a lower HU than water, it still exhibits a stable HU value from edge-to-edge. Moreover, beeswax could be used as a compensator if heated to 70°C. The HU of beeswax is less stable but still in an acceptable range.

Moreover, the phantom is made from a nonmagnetic material and might be applied to MR-based simulators or LINACs. The phantom with embedded fish oil capsules can perform image scan to evaluate the image quality of the grid pattern in MRI or MR-LINAC [13–15]. Silicone oil is a potential material for MRI phantoms [16]. A light curing technique with Al-Mg-Si alloys may be a better way to apply this test. However, these 3D-printed phantom slabs still need further improvements to make them suitable for various medical imaging systems. The patterns in the high-contrast-resolution slab can be printed using high-density plastic materials to make the high-contrast patterns visible for a CBCT system and reduce the interference caused by noise.

Based on the experimental results, both water and wax can be used for filling the uniformity slab. The HU values at the four edges meet the prescribed guideline within ±7 HU. However, silicon may not be a suitable material for field uniformity tests owing to the difficulty in evenly pouring silicone into the model, which causes significant variation in the HU at different edges. We plan to create a rod of the same material as the insert in the slab, and then fill it into the centre hole to observe the HU uniformity. The central rod will be filled with 1 cm (1.2 cm diameter) coins with the same perpendicular material. It is also feasible to fill the central rod with three sections of different materials to match the three materials in the uniformity phantom. To reduce artifacts caused by the hole, we plan to fill the beeswax or sealed water to improve our design in the future.

In this experiment, we aimed to find a less expensive and more stable material for CT uniformity testing. The results indicate that water and wax are two materials that can achieve stable HU values, and the HU difference is less than 5 when comparing each edge.

In further experiments, a light curing 3D printer could have the potential for further improvement of this phantom due to more dedicated printing. Partial parts of this phantom could be polished to achieve higher quality. We plan to create a rod of the same material as the insert in the slab, and then fill it into the centre hole to observe the uniformity test. The CT we used needs to apply an axial view scan to achieve the standard of ±5 HU. Almost all patients clinically received helical scan during simulation therefore the difference was higher than the ACR standard. Both the ACR standard and our phantom might be a way to solve this problem. The ACR scan has better homogeneity, but the phantom we offer has the advantages of being multifunctional and changeable. An institute could accumulate their own data by the ACR standard and our phantom initially and perform the QA procedure by our phantom thereafter to save valuable time.

## V. Conclusion

In conclusion, our results indicate that the homemade multifunctional phantom is suitable for both LINACs and CT simulators. Further measurements in an MR simulator and an MR-LINAC will be arranged in the future.
